# The relationship between antibody status to bovine corona virus and bovine respiratory syncytial virus and disease incidence, reproduction and herd characteristics in dairy herds

**DOI:** 10.1186/1751-0147-52-37

**Published:** 2010-06-04

**Authors:** Anna Ohlson, Ulf Emanuelson, Madeleine Tråvén, Stefan Alenius

**Affiliations:** 1 Department of Clinical Sciences, Swedish University of Agricultural Sciences, P.O. Box 7054, SE-750 07 Uppsala, Sweden

## Abstract

**Background:**

Bovine respiratory syncytial virus (BRSV) and bovine corona virus (BCV) affects cattle worldwide. Our objective was to evaluate the effects of these infections on general health and reproduction parameters measurable on herd level and to explore the association between antibody status and some herd characteristics.

**Methods:**

We collected a pooled milk sample from five primiparous cows from 79 Swedish dairy herds in September 2006. The samples were analysed for immunoglobulin G antibodies to BCV and BRSV with indirect enzyme-linked immunosorbent assays. Herd level data from 1 September 2005 to 30 August 2006 were accessed retrospectively. The location of the herds was mapped using a geographical information system.

**Results:**

Ten herds were antibody negative to both viruses and were compared with 69 herds positive to BCV or BRSV or both. Positive herds had a higher (P = 0.001) bulk tank milk somatic cell count (BMSCC) compared with negative herds. The medians for all other analyzed health and reproductive parameters were consistently in favour of the herds negative to both viruses although the differences were not statistically significant. A higher proportion (P = 0.01) of herds used professional technicians for artificial insemination, rather than farm personnel, amongst the 33 herds negative to BCV compared with the 46 positive herds.

**Conclusions:**

Our result shows that herds that were antibody positive to BCV and/or BRSV had a higher BMSCC compared with herds negative to BCV and BRSV. There was also tendency that negative herds had a better general herd health compared with positive. A higher proportion amongst the BCV negative herds used external technicians for AI instead of farm personnel, indicating that it is possible to avoid infection although having regular visits. Negative herds were located in close proximity to positive herds, indicating that local spread and airborne transmission between herds might not be of great importance and that herds can stay free from these infection transmission although virus is circulating in the area.

## Background

Bovine corona virus (BCV) and bovine respiratory syncytial virus (BRSV) are two worldwide distributed viruses [1,2]. BCV causes diarrhoea in calves, winter dysentery in adults and various degrees of respiratory symptoms [3-5]. BRSV is regarded as one of the most important causes of respiratory tract disease, especially in young calves. An infection can cause respiratory distress, fever, anorexia and subcutaneous emphysema and can lead to secondary bacterial pneumonia and death [6,7]. Outbreaks of BCV and BRSV occur mainly in autumn and winter [8,9]. These infections are common in dairy herds; in a nationwide survey in England and Wales the prevalence of antibodies to these viruses in bulk tank milk (BTM) was 100% [10]. Swedish studies have shown a prevalence of 70-100% for BCV and 41-89% for BRSV, with the higher prevalence in southern parts [7,11]. In a more recent study in a high animal-density area in south-west Sweden, the prevalence in BTM was 100% for both BCV and BRSV [12].

Previous studies have shown that BRSV and BCV infections are effectively spread within the herd [5,6,13]. It has also been shown that acquired antibodies remain detectable for years, even without reinfection [5,7], whereas maternal antibodies are only detectable for a few months. Spot samples from a few young animals can thus be used to reflect recent infections of BRSV and BCV in a herd, whereas bulk tank milk samples mirror the long-term history. Spot sampling has previously been described for bovine virus diarrea virus (BVDV) [14,15].

Despite the importance of these viruses and the fact that they are widely spread, little is known about transmission routes and management risk factors. Introduction of new animals and indirect spread via people and equipment are believed to be important and airborne transmission has been shown to occur for BRSV, at least under experimental conditions [16]. Studies have been carried out to determine the relationship between herd health, reproduction efficiency and milk production and seropositivity to other viruses, for example bovine viral diarrhoea virus and bovine leukemia virus [17-20]. Similar studies for BRSV and BCV have, as far as we know, not been conducted and it is therefore difficult to quantify their effect on the farm efficiency and economy. The purpose of this study was to explore if there were any associations between antibody status to BCV and BRSV and disease incidence, reproduction and some herd characteristics in dairy herds. A secondary aim was to investigate if there were any difference in proportion antibody positive herds between two neighbouring areas.

## Methods

### Study population and sampling

We used dairy herds from two neighbouring areas in central-eastern Sweden as study population. The areas approximately correspond to two veterinary districts. The herds were members of the local livestock association (Svenska Husdjur) and enrolled in the National Animal Disease Recording System (NADRS) [21] and the Swedish Official Milk Recording Scheme (SOMRS) [22]. All herds were free from BVDV according to the rules in the Swedish eradication program [23].

A convenience sample of 44 herds was taken from area 1 and of 35 herds from area 2, corresponding to 85% and 71% of the existing dairy herds in area 1 and 2, respectively. Sampling was performed by personnel from Svenska Husdjur. Herds were included if the farmer agreed to participate in the study, in the order they were visited by the personnel. The sampling period was from September 1^st ^to October 31^st ^2006. The geographical locations of the herds were mapped using a geographic information system (ArcGIS, 2005).

A pooled milk sample from five home-bred primiparous cows was collected from each herd [24]. We used 10-ml test tubes containing 1.5 mg of the preservative agent Bronopol (2-bromo-2-nitropropane-1.3-diol). The milk samples were stored at -20°C until analysis.

### Antibody detection and cut off

The milk samples were analysed for presence of immunoglobulin G antibodies to BCV [5] and BRSV [25] by commercially available indirect enzyme-linked immunosorbent assays (ELISA; Svanova Biotech). The same batch was used for all analyses. The samples were not diluted or centrifuged. The optical density (OD) at 450 nm was corrected by subtraction of the negative control antigen OD. To adjust for day-to-day variations we calculated the percent positivity (PP) as (corrected OD/positive control corrected OD) × 100. A PP-value of <20 was regarded as negative for the pooled samples, closely corresponding to the corrected OD of 0.20 which is the cut off for negative individual milk, both for BCV and BRSV, recommended by the manufacturer.

### Outcome variables

Herd level data on number of cows, AI-strategy, milk production, reproductive performance and health status from September 1^st ^2005 to August 31^st ^2006 were obtained from NADRS and SOMRS. Disease incidences included treated cases of mastitis, any treatment for fertility problems, diseases in young stock and udder disease score (UDS) ≥6. UDS describes the udder health based on individual SCC corrected for milk yield, breed, number of parity and time in lactation [26]. The scale is from 0 to 9 and expresses the probability that a cow has mastitis. UDS class ≥6 implies a 60% probability of infection and corresponds to having an SCC of approximately ≥300 000 cells/ml. Mastitis, treatment for fertility problems, culling and diseases in young stock were calculated as the yearly incidence rates (IR), i.e. number of cases divided by number of cow days or young stock days. Early and late calf mortality was calculated as the mean of 12 monthly IR; for which each monthly IR was calculated as number of calves dead at birth or within 24 hours divided by number of calvings, and number of calves dead between 1 day and 3 months of age divided by number of calves surviving 24 hours, respectively. The incidence of UDS ≥6 was calculated as a daily mean, i.e. number of cases with UDS ≥6 divided by number of cow days. Milk yield was expressed as the mean milk yield per cow and year in kg and BMSCC as the geometric mean of 12 monthly measurements. The data on reproductive performance included average calving interval, time from calving to first insemination and number of inseminations per service period.

### Explanatory variables

The main predictor was antibody status, defined as NEG for herds that were antibody negative to both BRSV and BCV, and POS for herds positive to either BRSV or BCV or both, based on the result from the pooled milk samples. Herd characteristics were also compared separately for BCV and BRSV. The models also included the effects of herd size, milk production, breed and AI-strategy according to table 1. Herd size and milk production was dichotomized by the median, 43 cows and 8967 kg milk per cow and year respectively. In 2006 the mean herd size in Sweden was 48 cows and the mean milk yield was 8175 kg per cow and year [27]. Breed was classified into three categories: > 80% Swedish Red and White breed (SRB), > 80% Swedish Holstein breed (SH) and mixed/other breeds. SRB and SH are the two main dairy-cow breeds in Sweden. Finally, AI-strategy was defined as insemination performed by farm personnel (AIF) or by professional technicians (AIT).

**Table 1:** Statistical models used to study associations between outcome variables and herd antibody status

*Outcome variable*	*Data type*	*Model type*	***Explanatory variables***^***1***^
Milk yield	Continuous	Linear	a, b, n
BTMSCC^2^	Continuous	Linear	a, b, n, p
Calving interval	Continuous	Linear	a, b, n, p, i
Calving to first AI^3^	Continuous	Linear	a, b, n, p, i
Number of AI^3^	Continuous	Linear	a, b, n, p, i
Dead calves 0-24 hours	Binomial	Logistic	a, b, n
Culling	Binomial	Logistic	a, b, n, p
UDS^4 ^≥6	Binomial	Logistic	a, n

^1 ^a = antibody status, b = breed, n = herd size, p = milk production level, i = artificial insemination strategy

^2 ^bulk tank milk somatic cell count.

^3 ^artificial insemination

^4 ^udder disease score, see main text for explanation.

### Statistical analysis

We used t-test to compare herd size and Fisher's exact test to compare breed and AI strategy between NEG and POS herds. Fisher's exact test was also used to compare the proportion of antibody positive herds between the areas.

The outcome variables mastitis, treatment for fertility problems, number of calves dead between one day and three months and diseases in young stock had more than 10% missing values and were not analysed because the remaining sample of NEG herds was considered too small. The continuous outcome variables were analysed with a linear regression model whereas logistic regression models were used for the binomial outcome variables. The eight models are shown in Table 1. We chose to keep the models intact because we considered the predictor variables included in each model as biologically important for the outcome. In order to achieve normal distribution of the residuals we transformed number of AI and calving interval to the natural logarithmic scale (ln). BMSCC was transformed by taking the square root before analysis.

The predictor variables were tested for collinearity by pair-wise Spearman rank correlation test; a test result of <0.6 was considered as negative i.e. no strong correlation. There were no collinearities found between the explanatory variables. No interactions were tested in the statistical model because of the small sample size. To evaluate the fit of the models with significant outcome we plotted the studentised residuals against the predicted observation. The statistical analyses were done using Stata Software (StataCorp. 2006; Stata Statistical Software: Release 9.0; College Station, TX, USA: StataCorp LP).

## Results

### Study population and immunity

**Figure 1:**
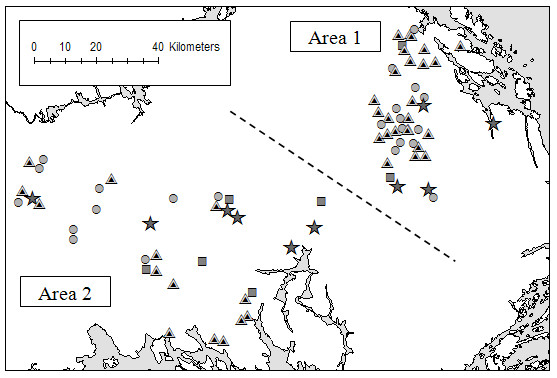
**Geographical distribution**. Geographical distribution of 79 Swedish dairy herds, stratified by antibody-status to bovine coronavirus (BCV) and bovine respiratory syncytial virus (BRSV) as measured by an ELISA test in pooled milk samples of 5 primiparous cows sampled in September-October 2006. *Star = Negative for BRSV and BCV Circle = Positive for BRSV, negative for BCV Square = Negative for BRSV, positive for BCV Triangle = Positive for BRSV and BCV*

Number of herds that were antibody positive in the pooled milk samples was for BCV 46 (58.2%) and for BRSV 62 (78.5%). Ten herds were classified as NEG and 69 herds as POS The proportion of antibody positive herds did not differ significantly between the two areas under study for both BCV and BRSV (table 2). Geographical locations of the herds stratified by antibody status are shown in figure 1.

**Table 2:** Antibody status to bovine respiratory syncytial virus (BRSV) and bovine coronavirus (BCV)

	Number of herds (percent)			
		
BRSV	BCV	Area 1, n = 44	Area 2, n = 35	All, n = 79
-	-	4 (9.1)	6 (17.1)	10 (12.6)
+	-	13 (29.5)	10 (28.6)	23 (29.1)
-	+	2 (4.5)	5 (14.3)	7 (8.9)
+	+	25 (56.8)	14 (40.0)	39 (49.4)

Antibody status of 79 Swedish dairy herds in two neighbouring areas based on pooled milk samples from five home-bred primiparous cows, measured by an ELISA and collected in September-October 2006

### Analyses of herd data

The NEG group had a lower mean BMSCC (P < 0.001) compared with the POS group. No other significant (P > 0.05) differences were found between NEG and POS herds in the remaining outcome variables. The median and interquartile range of each variable is presented in table 3.

**Table 3:** Summary of the analysed variables

	NEG herds		POS herds	
	
Variable	Median	IQR	Median	IQR
Herd size, cow-years	57	40-75	43	32-62
Milk yield, kg/cow-year	9013	8640-9982	8964	88315-8759
BTMSCC^1^, 1000 cells/ml	163	140-187	218	164-283
Calving interval, days	390	381-413	402	387-415
Calving to first AI^2^, days	84	76-98	91	80-104
Number of AI^2^	1.7	1.5-2.3	1.8	1.6-2
Dead calves^3 ^0-24 h	0.040	0.033-0.052	0.047	0.028-0.093
Culling^3^	0.26	0.23-0.39	0.36	0.26-0.42
UDS^3,4 ^≥ 6	1.3	0.97-1.4	1.7	1.2-2.3

Comparison between 10 Swedish dairy herds antibody negative (NEG) to bovine coronavirus (BCV) and bovine respiratory syncytial virus (BRSV) and 69 herds positive (POS) to either BRSV or BCV or both, measured by an ELISA test in pooled milk samples from five home-bred primiparous cows, collected in September-October 2006.

^1 ^Bulk tank milk somatic cell count, geometric mean of 12 monthly measurements. P < 0.001 estimated in a linear regression model.

^2 ^AI = artificial insemination

^3 ^One-year incidence rate

^4 ^UDS = udder disease score, see main text for explanation.

Herd size, milk production, breed and AI strategy did not differ significantly between the POS and NEG herds. Of all sampled herds 72% used AIT; 80% in NEG group and 71% in the POS group. There was a significantly (P < 0.01) higher proportion using AIT (29/33) amongst the herds negative to BCV, compared with the proportion amongst BCV positive herds (28/46). For BRSV, 12 out of 17 negative herds used AIT; corresponding numbers for the positive herds was 45 out of 62. For the distribution of breed, 56% of all herds had mainly SRB, 4% SHB and 40% had mixed/other breeds.

## Discussion

The only herd health parameter with statistically significant differences between POS and NEG herds was BMSCC for which POS herds had a higher BMSCC than NEG herds. Despite the difficulties to evaluate causality in cross-sectional studies it is notable that, although not statistically significant, the medians for all analysed variables were consistently in favour of the NEG herds. One explanation for the lower BMSCC in NEG herds may be that an outbreak of BCV or BRSV increases the susceptibility to clinical or subclinical udder infections. Another explanation can be that these herds are better managed with better hygienic routines also at cow level. The information on mastitis incidence had too many missing values to be analyzed. The incidence of high UDS pointed in the same direction as the BMSCC, although the difference was not statistically significant. A high BMSCC is of economic importance for the farmers because the dairy (Arla foods in this area) pays 1% extra for milk with a cell count of ≤300 000 cells/ml and 2% extra when SCC is ≤200 000/ml whereas a deduction of 4% is made for milk with a cell count of ≥401 000 cells/ml and 10% for ≥501 000 cells/ml. Studies have also shown that a high SCC is related to a loss in milk yield [28-30] and that BMSCC is correlated with prevalence of subclinical mastitis [31]. The tendency to poorer reproductive performance and higher culling rate amongst the POS herds could be a consequence of the disease outbreaks by for example delaying AI. These findings may also be related to better general herd management.

A surprising result was that a higher proportion of herds used professional AI technicians rather than AI performed by farm personnel amongst the herds negative to BCV compared with the herds positive to BCV. This finding confirms the results the study by Bidokhti et al [32] where AI-technicians were more common in antibody negative herds compared to positive regarding both BCV and BRSV. Having visitors should not be a protective factor, especially persons that visit more than one herd each day. For example; a typically 50-cow AIT herd in study area 1 and 2 has two AI-technician visits per week, and each AI-technician in these areas visits approximately six herds per day (Rose-Marie Winroth, personal communication). An explanation could be that technicians educate the farmers continuously in how to protect their herd against contagious diseases, thus keeping updated routines in handing protective clothing for visitors as well as providing cleaning and disinfection possibilities. Another explanation could be that these farmers manage the herd more efficiently and therefore use professionals for AI instead of doing it themselves, and well managed herds are also more likely to avoid infection. In all cases, our result indicates clearly that it is possible to avoid infection even with regular visitors in the herd.

Several negative herds were located in close proximity to positive herds, e.g. one of the NEG herds was located between two herds positive to both viruses, at a distance of 1.3 km and 1.9 km. This indicates that local spread and airborne transmission between herds are not of great importance for these infections and that a herd can stay negative although virus is circulating in the area.

The cows included in the pooled milk sample were primiparous, which means that positive herds have experienced an infection during the last two years, approximately. Some herds in the POS group could have had the infection in the year before the investigated year, which may lead to smaller differences between NEG and POS herds. Herds in the NEG group, however, have been free from infection during the year under investigation.

There is a risk of selection bias because the herds were selected by personnel from the local livestock association and only farmers willing to participate were included in the study. The antibody status of the herds was, however, not known at the time of sampling. The disease and reproduction data in the NADRS and SOMRS are reported by farmers, AI technicians and veterinarians. The reports from veterinarian and technicians should not differ between the study groups, but there could be differences in the willingness to report amongst the farmers causing information bias. It is possible that well managed herds with high biosecurity are also better in reporting, which may lead to higher incidences of diseases in NEG herds and thus an underestimate of the differences between the groups. BMSCC, UDS and milk yield are objective measurements from the dairies and from the milk-recording schemes and are not influenced by information bias.

It would be of interest to further investigate the association between BCV/BRSV infections and herd health parameters on a larger scale. It would be beneficial to follow herds over time and compare uninfected herds with herds that have shown primary infection during the study period.

## Conclusion

In this study we found that herds that were antibody positive to BCV and/or BRSV had a higher BMSCC compared with herds negative to BCV and BRSV. There was a tendency that negative herds had a better general herd health compared with positive. A higher proportion amongst the BCV negative herds used external technicians for AI instead of farm personnel, indicating that it is possible to avoid infection although having regular visits. Negative herds were located geographically close to positive, indicating that local spread and airborne transmission between herds is not the major transmission routes for BCV and BRSV infections.

## Competing interests

The authors declare that they have no competing interests.

## Authors' contributions

AO carried out the statistical analysis and drafted the manuscript. UE assisted the statistical analysis. UE, MT and SA conceived the study. All authors participated in its design and coordination, were involved in revising the manuscript and read and approved the final manuscript.

## References

[B1] Clark MA Bovine coronavirus Br Vet J 1993 149 51 70 8382546 10.1016/S0007-1935(05)80210-6 PMC7130254

[B2] Valarcher JF Taylor G Bovine respiratory syncytial virus infection Vet Res 2007 38 153 180 10.1051/vetres:2006053 17257568

[B3] Stair EL Rhodes MB White RG Mebus CA Neonatal calf diarrhea: purification and electron microscopy of a coronavirus-like agent Am J Vet Res 1972 33 1147 1156 4553881

[B4] Saif LJ A review of evidence implicating bovine coronavirus in the etiology of winter dysentery in cows: an enigma resolved? Cornell Vet 1990 80 303 311 2170075

[B5] Alenius S Niskanen R Juntti N Larsson B Bovine coronavirus as the causative agent of winter dysentery: serological evidence Acta Vet Scand 1991 32 163 170 1666489 10.1186/BF03546976 PMC8127893

[B6] Verhoeff J Van der Ban M van Nieuwstadt AP Bovine respiratory syncytial virus infections in young dairy cattle: clinical and haematological findings Vet Rec 1984 114 9 12 6702077 10.1136/vr.114.1.9

[B7] Elvander M Severe respiratory disease in dairy cows caused by infection with bovine respiratory syncytial virus Vet Rec 1996 138 101 105 8650902 10.1136/vr.138.5.101

[B8] Stott EJ Thomas LH Collins AP Crouch S Jebbett J Smith GS Luther PD Caswell R A survey of virus infections of the respiratory tract of cattle and their association with disease J Hyg (Lond) 1980 85 257 270 6256435 10.1017/s0022172400063294 PMC2133932

[B9] Van der Poel WH Kramps JA Middel WG Van Oirschot JT Brand A Dynamics of bovine respiratory syncytial virus infections: a longitudinal epidemiological study in dairy herds Arch Virol 1993 133 309 321 10.1007/BF01313771 8257292

[B10] Paton DJ Christiansen KH Alenius S Cranwell MP Pritchard GC Drew TW Prevalence of antibodies to bovine virus diarrhoea virus and other viruses in bulk tank milk in England and Wales Vet Rec 1998 142 385 391 9586130 10.1136/vr.142.15.385

[B11] Tråvén M Björnerot L Larsson B Nationwide survey of antibodies to bovine coronavirus in bulk milk from Swedish dairy herds Vet Rec 1999 144 527 529 10378280 10.1136/vr.144.19.527

[B12] Hägglund S Hjort M Graham DA Ohagen P Törnquist M Alenius S A six-year study on respiratory viral infections in a bull testing facility Vet J 2007 173 585 93 10.1016/j.tvjl.2006.02.010 16647871 PMC7110487

[B13] Hägglund S Svensson C Emanuelson U Valarcher JF Alenius S Dynamics of virus infections involved in the bovine respiratory disease complex in Swedish dairy herds Vet J 2006 172 320 328 10.1016/j.tvjl.2005.04.029 15964774 PMC7110557

[B14] Houe H Serological analysis of a small herd sample to predict presence or absence of animals persistently infected with bovine viral diarrhoea virus (BVDV) in dairy herds Res Vet Sci 1992 53 320 323 1334566

[B15] Houe H Bovine virus diarrhoea virus: detection of Danish dairy herds with persistently infected animals by means of a screening test of ten young stock Preventive Veterinary Medicine 1994 19 241 248 10.1016/0167-5877(94)90092-2

[B16] Mars MH Bruschke CJ van Oirschot JT Airborne transmission of BHV1, BRSV, and BVDV among cattle is possible under experimental conditions Vet Microbiol 1999 66 197 207 10.1016/S0378-1135(99)00009-7 10227122

[B17] Tiwari A VanLeeuwen JA Dohoo IR Stryhn H Keefe GP Haddad JP Effects of seropositivity for bovine leukemia virus, bovine viral diarrhoea virus, Mycobacterium avium subspecies paratuberculosis, and Neospora caninum on culling in dairy cattle in four Canadian provinces Vet Microbiol 2005 109 147 158 10.1016/j.vetmic.2005.05.011 15970402

[B18] Emanuelson U Scherling K Pettersson H Relationships between herd bovine leukemia virus infection status and reproduction, disease incidence, and productivity in Swedish dairy herds Preventive Veterinary Medicine 1992 12 121 131 10.1016/0167-5877(92)90075-Q

[B19] Niskanen R Emanuelson U Sundberg J Larsson B Alenius S Effects of infection with bovine virus diarrhoea virus on health and reproductive performance in 213 dairy herds in one county in Sweden Preventive Veterinary Medicine 1995 23 229 237 10.1016/0167-5877(94)00437-N

[B20] Berends IM Swart WA Frankena K Muskens J Lam TJ van Schaik G The effect of becoming BVDV-free on fertility and udder health in Dutch dairy herds Prev Vet Med 2008 84 48 60 Epub 2007 Dec 2026 10.1016/j.prevetmed.2007.11.002 18155307

[B21] Emanuelson U The national Swedish animal disease recording system Acta Vet Scand Suppl 1988 84 262 264 3232620

[B22] Olsson SO Baekbo P Hansson SO Rautala H Osteras O Disease recording systems and herd health schemes for production diseases Acta Vet Scand Suppl 2001 94 51 60 10.1186/1751-0147-42-S1-S51 11875853 PMC8041038

[B23] Lindberg AL Alenius S Principles for eradication of bovine viral diarrhoea virus (BVDV) infections in cattle populations Vet Microbiol 1999 64 197 222 10.1016/S0378-1135(98)00270-3 10028173

[B24] Ohlson A Tråvén M Emanuelson U Alenius S The relationship between pooled and individual milk samples for detecting antibodies to bovine coronavirus and bovine respiratory syncytial virus12th Symposium of the International Society for Veterinary Epidemiology and Economics August 10-14, 2009 2009 Durban, South Africa

[B25] Elvander M Edwards S Näslund K Linde N Evaluation and application of an indirect ELISA for the detection of antibodies to bovine respiratory syncytial virus in milk, bulk milk, and serum J Vet Diagn Invest 1995 7 177 182 7619898 10.1177/104063879500700202

[B26] Brolund L Technical utilization of cell count in the milk-recording service (Cellhaltens tekniska utnyttjande i kokontrollen). Pages 40-41 in Djurhälsovård 88/89. Meddelande nr 161. Swedish Association for Livestock Breeding and Production 1990 Eskilstuna, Sweden (in Swedish)

[B27] (Svensk Mjölk), Retrieved 07/11/15, from http://www.svenskmjolk.se

[B28] Bennedsgaard TW Enevoldsen C Thamsborg SM Vaarst M Effect of mastitis treatment and somatic cell counts on milk yield in Danish organic dairy cows J Dairy Sci 2003 86 3174 3183 10.3168/jds.S0022-0302(03)73920-4 14594237

[B29] Losinger WC Economic impacts of reduced milk production associated with an increase in bulk-tank somatic cell count on US dairies J Am Vet Med Assoc 2005 226 1652 1658 10.2460/javma.2005.226.1652 15906563

[B30] Green LE Schukken YH Green MJ On distinguishing cause and consequence: do high somatic cell counts lead to lower milk yield or does high milk yield lead to lower somatic cell count? Prev Vet Med 2006 76 74 89 Epub 2006 Jun 2014 10.1016/j.prevetmed.2006.04.012 16780974

[B31] Lukas JM Hawkins DM Kinsel ML Reneau JK Bulk tank somatic cell counts analyzed by statistical process control tools to identify and monitor subclinical mastitis incidence J Dairy Sci 2005 88 3944 3952 10.3168/jds.S0022-0302(05)73080-0 16230700

[B32] Bidokhti MR Traven M Fall N Emanuelson U Alenius S Reduced likelihood of bovine coronavirus and bovine respiratory syncytial virus infection on organic compared to conventional dairy farms Vet J 2009 182 436 440 10.1016/j.tvjl.2008.08.010 18835795 PMC7110579

